# Supplementary material for: Shared trans-ancestry architecture of HLA-mediated disease risk in the All of Us Research Program
Source: medRxiv. 2026 Jun 30:2026.06.26.26356709. Preprint. [Version 1] doi: 10.64898/2026.06.26.26356709 (PMC13345497; doi:10.64898/2026.06.26.26356709)
Supplement: 1 [file NIHPP2026.06.26.26356709V1-supplement-1.pdf]

medRxiv preprint doi: https://doi.org/10.64898/2026.06.26.26356709; this version posted June 30, 2026. The copyright holder for this preprint (which was not certified by peer review) is the author/funder, who has granted medRxiv a license to display the preprint in perpetuity. This article is a US Government work. It is not subject to copyright under 17 USC 105 and is also made available for use under a CC0 license.

Supplementary Table 1. Cohort sizes for WGS analyses after removal of related individuals

AFR	AMR	EAS	EUR	MID	SAS	Total
77657	72151	9675	224559	1458	5323	390823

Number of unrelated participants included in HLA diversity and linkage disequilibrium analyses after exclusion of related individuals (identity-by-descent > 0.2). AFR, African; AMR, Admixed American; EAS, East Asian; EUR, European; MID, Middle Eastern; SAS, South Asian.

medRxiv preprint doi: https://doi.org/10.64898/2026.06.26.26356709; this version posted June 30, 2026. The copyright holder for this preprint (which was not certified by peer review) is the author/funder, who has granted medRxiv a license to display the preprint in perpetuity. This article is a US Government work. It is not subject to copyright under 17 USC 105 and is also made available for use under a CC0 license.

Supplementary Figure 1. Expected heterozygosity across HLA loci and ancestry groups.

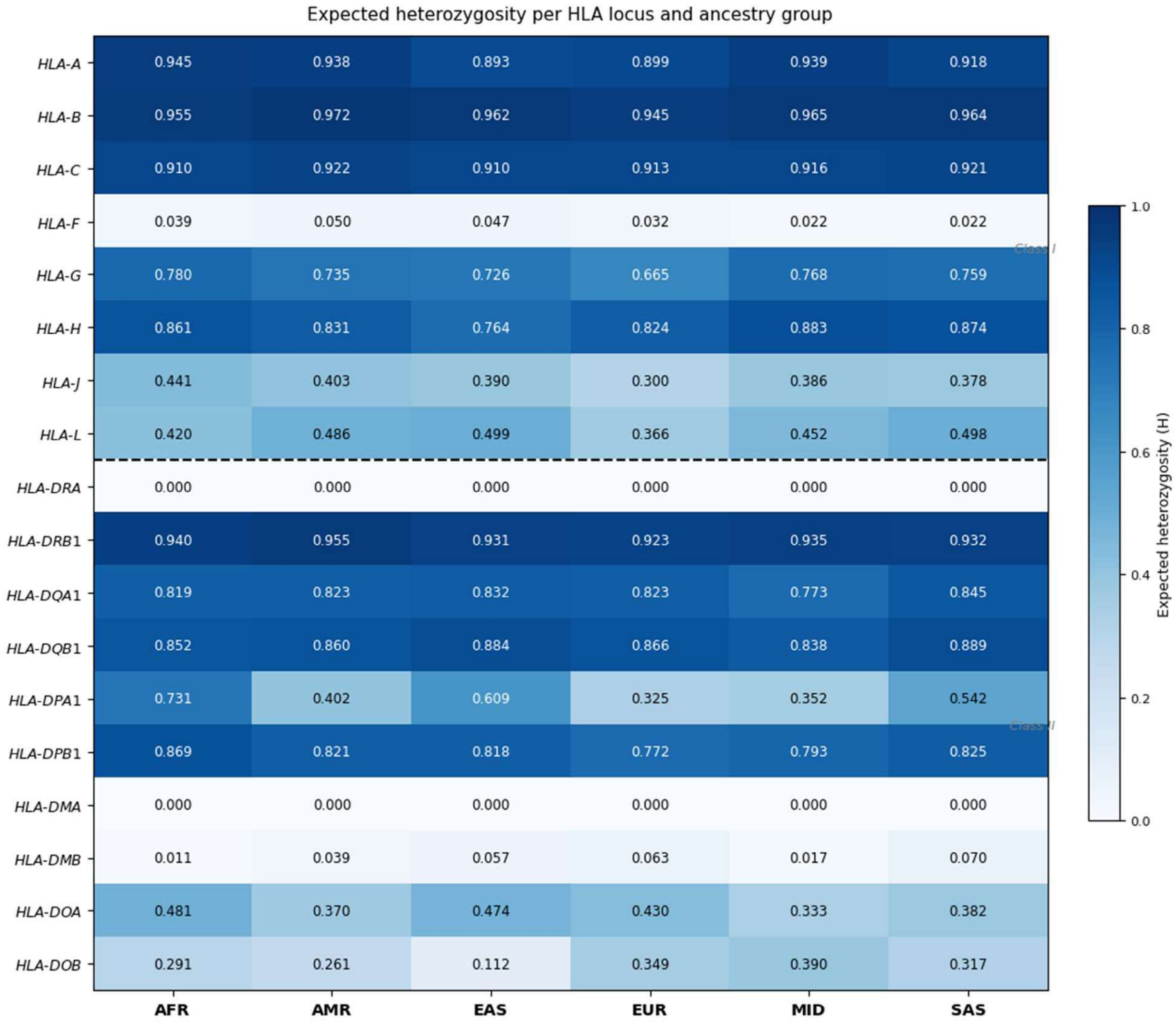

Heatmap showing expected heterozygosity ($H = 1 - \Sigma p_i^2$) calculated from allele frequencies across HLA loci and ancestry groups. Higher values indicate greater allelic diversity. HLA-DRB3 and HLA-DRB5 were excluded because of haplotype-dependent gene presence.

medRxiv preprint doi: https://doi.org/10.64898/2026.06.26.26356709; this version posted June 30, 2026. The copyright holder for this preprint (which was not certified by peer review) is the author/funder, who has granted medRxiv a license to display the preprint in perpetuity.
This article is a US Government work. It is not subject to copyright under 17 USC 105 and is also made available for use under a CC0 license.

Supplementary Figure 2. LD among independent conditional HLA alleles across ancestries

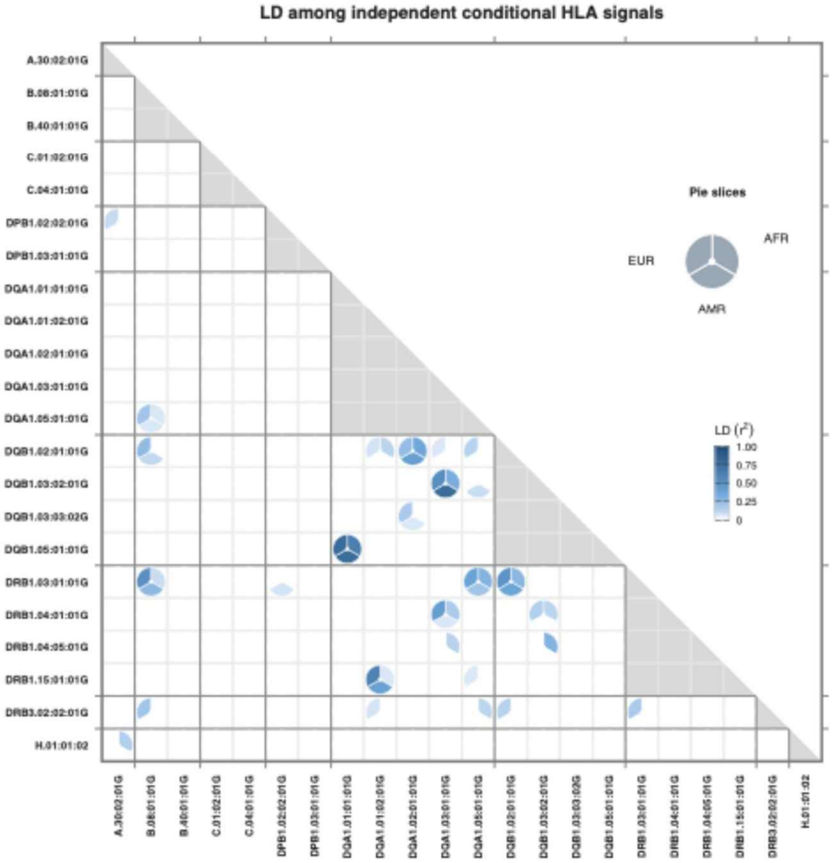

Heatmap of inter-locus allele-level linkage disequilibrium (LD; r^2) among 22 HLA alleles identified by stepwise conditional meta-analysis across five phenotypes. Alleles are ordered by gene, with black divider lines indicating gene boundaries. Within-gene comparisons (including the diagonal) are shown in light gray. Increasingly darker shades of blue indicate stronger LD.
